# EASI Transformation: An Efficient Transient Expression Method for Analyzing Gene Function in *Catharanthus roseus* Seedlings

**DOI:** 10.3389/fpls.2019.00755

**Published:** 2019-06-11

**Authors:** Samuel Mortensen, Diana Bernal-Franco, Lauren F. Cole, Suphinya Sathitloetsakun, Erin J. Cram, Carolyn W. T. Lee-Parsons

**Affiliations:** ^1^Department of Biology, Northeastern University, Boston, MA, United States; ^2^Department of Chemical Engineering, Northeastern University, Boston, MA, United States; ^3^Department of Bioengineering, Northeastern University, Boston, MA, United States; ^4^Department of Chemistry and Chemical Biology, Northeastern University, Boston, MA, United States

**Keywords:** *Catharanthus roseus*, agroinfiltration, transient transformation, transactivation, ORCA3, ZCT1, mutated *VirG*, viral silencing suppressors

## Abstract

The *Catharanthus roseus* plant is the exclusive source of the valuable anticancer terpenoid indole alkaloids, vinblastine (VB) and vincristine (VC). The recent availability of transcriptome and genome resources for *C. roseus* necessitates a fast and reliable method for studying gene function. In this study, we developed an *Agrobacterium*-mediated transient expression method to enable the functional study of genes rapidly *in planta*, conserving the compartmentalization observed in the VB and VC pathway. We focused on (1) improving the transformation method (syringe versus vacuum agroinfiltration) and cultivation conditions (seedling age, *Agrobacterium* density, and time point of maximum transgene expression), (2) improving transformation efficiency through the constitutive expression of the virulence genes and suppressing RNA silencing mechanisms, and (3) improving the vector design by incorporating introns, quantitative and qualitative reporter genes (luciferase and *GUS* genes), and accounting for transformation heterogeneity across the tissue using an internal control. Of all the parameters tested, vacuum infiltration of young seedlings (10-day-old, harvested 3 days post-infection) resulted in the strongest increase in transgene expression, at 18 – 57 fold higher than either vacuum or syringe infiltration of other seedling ages. Endowing the *A. tumefaciens* strain with the mutated *VirGN54D* or silencing suppressors within the same plasmid as the reporter gene further increased expression by 2 – 10 fold. For accurate measurement of promoter transactivation or activity, we included an internal control to normalize the differences in plant mass and transformation efficiency. Including the normalization gene (*Renilla* luciferase) on the same plasmid as the reporter gene (firefly luciferase) consistently yielded a high signal and a high correlation between RLUC and FLUC. As proof of principle, we applied this approach to investigate the regulation of the *CroSTR1* promoter with the well-known activator ORCA3 and repressor ZCT1. Our method demonstrated the quantitative assessment of both the activation and repression of promoter activity in *C. roseus*. Our efficient *Agrobacterium*-mediated seedling infiltration (EASI) protocol allows highly efficient, reproducible, and homogenous transformation of *C. roseus* cotyledons and provides a timely tool for the community to rapidly assess the function of genes *in planta*, particularly for investigating how transcription factors regulate terpenoid indole alkaloid biosynthesis.

## Introduction

Plants produce a vast array of specialized metabolites that enable adaptation to environmental challenges. For instance, plants produce compounds that can attract pollinators or deter pathogens and herbivores (Weng et al., 2012). For humans, these specialized metabolites are an important source of useful chemicals, particularly medicines (Balunas and Kinghorn, 2005; Wurtzel and Kutchan, 2016).

During the past five years, the availability of transcriptome and genome resources for medicinal plants has grown substantially; examples include the Medicinal Plant Genomics Resource^1^, PhytoMetaSyn^2^, and 1000 Plants Initiative^3^. In addition, advances in metabolomic technologies now enable single-cell and spatial metabolite profiling (Wurtzel and Kutchan, 2016). These approaches are accelerating the identification of candidate genes important for the biosynthesis of these medicinal compounds and for the regulation of their biosynthetic genes. There is a timely need for the functional study of this growing list of genes. For this reason, we developed a fast and reliable method to study gene function in the medicinal plant, *Catharanthus roseus* (L.) G. Don (van der Heijden et al., 2004; Facchini and De Luca, 2008).

*Catharanthus roseus* produces two potent anticancer terpenoid indole alkaloids (TIA), vinblastine and vincristine, which were serendipitously discovered in the 1950s while investigating the plant’s reputation for the treatment of diabetes (Noble, 1990). Since the anticancer TIAs are only found in trace amounts in *C. roseus* leaves (Andersson et al., 1996), they are the target of production by biotechnological means (van der Heijden et al., 2004). The first comprehensive transcriptome resource for *C. roseus* was released in 2012 (Góngora-Castillo et al., 2012) and the first partial genome in 2015 (Kellner et al., 2015). The availability of these and other resources enabled the pathway for catharanthine and vindoline biosynthesis, the two precursors to the anticancer compounds vinblastine and vincristine, to be fully elucidated (Qu et al., 2015, 2018; Caputi et al., 2018). The pathway involves 28 known enzymes distributed across several cell types in the leaf including internal phloem-associated parenchyma cells, epidermis, laticer/idioblasts, and different subcellular compartments (reviewed in Pan et al., 2016). Multiple transcription factors and transporter genes involved in trafficking of the pathway intermediates across compartments have also been identified (Menke et al., 1999; Pasquali et al., 1999; van der Fits and Memelink, 2000; Chatel et al., 2003; Pauw et al., 2004; Suttipanta et al., 2011; Zhang et al., 2011; Yu and De Luca, 2013; Van Moerkercke et al., 2015, 2016; Paul et al., 2017; Payne et al., 2017; Patra et al., 2018). Previous attempts to increase alkaloid production by expressing single pathway genes or altering the expression of transcription factors have resulted in limited success, suggesting the importance of investigating the complex regulation underlying their biosynthesis (Rizvi et al., 2016; Schweizer et al., 2018; Sun et al., 2018; reviewed in Pan et al., 2016). Tools are needed to rapidly assess the regulation of catharanthine and vindoline biosynthesis in appropriate tissue type, toward engineering increased production in the future.

Methods to evaluate gene function are limited in *C. roseus* compared to *Arabidopsis thaliana*, tobacco, and other model plants. As in other plants, development of transgenic plants via regeneration from tissue culture is time-consuming, expensive, and genotype-dependent (Zhao et al., 2017). Few reports exist regarding the development of transgenic plants in *C. roseus* (Pan et al., 2012; Wang et al., 2012). In *C. roseus*, the development of transgenic hairy roots is commonly pursued because an efficient and reliable method exists (Choi et al., 2004; Rizvi et al., 2015). However, the disadvantage of using hairy roots is that many genes of the TIA pathway are regulated differently in roots than leaves (reviewed in Pan et al., 2016), and hairy roots require 4–6 months for development. Therefore, the generation of hairy root lines is unsuitable for quick screening of leaf-specific candidate genes.

For evaluating gene function more rapidly, model plants like tobacco have been used for promoter transactivation studies of *C. roseus* genes (Van Moerkercke et al., 2015, 2016; Paul et al., 2017; Patra et al., 2018; Schweizer et al., 2018). However, the vindoline pathway is exclusively found in *C. roseus*. Therefore, the regulation of the vindoline pathway, including monitoring expression and metabolite levels, needs to be studied in *C. roseus* tissue. Initial attempts to transiently transform *C. roseus* were successfully made in calli, cell suspensions, and protoplasts using methods such as biolistic transformation, *Agrobacterium*, or electroporation (van der Fits and Memelink, 1997; Guirimand et al., 2009; Suttipanta et al., 2011). However, these systems lack the specialized cells or subcellular compartmentalization observed in cotyledons and leaves where vindoline biosynthesis predominantly occurs (Aerts et al., 1994; Pan et al., 2016). Other drawbacks of biolistic or protoplast transformations include low transformation rates, the requirement of specialized equipment, and extensive optimization. Subsequent transient transformation attempts in *C. roseus* have focused on *Agrobacterium*-mediated transient transformation of detached leaves (Di Fiore et al., 2004), petals (Van Moerkercke et al., 2016, adapted from Verweij et al., 2008), and seedlings (Makhzoum et al., 2011; Weaver et al., 2014). However, we observed low transformation efficiency in mature tissue (unpublished data). Additionally, the petal transient transformation method requires flowers harvested from plants grown under special photoperiods to induce flowering year-round in a greenhouse. Of these tissues, seedlings are ideal because they have intact plant tissue structure, are obtained in a matter of days, and can be cultivated in tissue culture boxes in the laboratory.

In this study, we developed a transient expression method in *C. roseus* seedlings to enable functional study of genes rapidly *in planta*, conserving the compartmentalization observed in the vindoline pathway. We completely revised our *Agrobacterium*-mediated transient expression method (Weaver et al., 2014) by focusing on three main aspects: (1) improving throughput (syringe versus vacuum infiltration) and cultivation conditions (seedling age, *Agrobacterium* density, and time point of maximum transgene expression), (2) improving transformation efficiency through the constitutive expression of the virulence genes (via the constitutively active VirG) and suppressing RNA silencing mechanisms (via viral silencing suppressors), and (3) improving the vector design by incorporating introns, quantitative and qualitative reporter genes (luciferase and *GUS* genes), and accounting for transformation heterogeneity across the tissue using an internal control.

We developed this quantitative transient expression assay for studying the regulation underlying TIA biosynthesis. For this application, the firefly and *Renilla* luciferase reporter genes were selected due to their sensitivity, dynamic range, and time-resolution for the accurate measurement of transcription factor and promoter activity. As proof of principle, we applied our transient expression assay to demonstrate the transactivation of the *Strictosidine synthase* (*STR1*) promoter by the Octadecanoid-Responsive *Catharanthus* AP2 (ORCA3) transcriptional activator (van der Fits and Memelink, 2001) and by a repressor from the Zinc finger protein family of *Catharanthus* (ZCT1) (Pauw et al., 2004). Overall, we developed a reproducible, robust, and highly efficient transient expression method, known as efficient *Agrobacterium*-mediated seedling infiltration (EASI) protocol, for studying gene function in *C. roseus* seedlings. This low-cost method requires no specialized equipment and offers high transformation rates, high throughput, and rapid generation of results.

## Materials and Methods

### Preparation of *C. roseus* Seedlings

*Catharanthus roseus* seeds were surface-sterilized by submerging 0.8 g of seeds (Little Bright Eye, NEseeds) in 20 ml of 5% PPM (Plant Preservative Mixture, Caisson Laboratory) for 16 h at 25°C in the dark (day 0). After discarding the 5% PPM, the seeds were spread evenly on top of full-strength Gamborg’s media (3.1 g/L of Gamborg’s basal salts, 1 ml/L of Gamborg’s vitamins (1000×), 6% micropropagation agar type 1, Phytotechnology Laboratory), inside a sterile Magenta^TM^ box (Sigma) (day 1). Seeds were kept in the dark at 25°C for 7 days when most seeds germinated and were at the hook stage (i.e., seedlings had the cotyledons pointing downward and hypocotyl was around 1 cm long). On day 8, the seedlings in the Magenta boxes were then transferred to 16 h of light (Erligpowht 45W LED Red Blue Lights) and 8 h of dark at room temperature for 2 days (except in section “Optimum Transgene Expression Occurs With Vacuum Infiltration of Young Seedlings” with the optimization of seedling age). On day 10, seedlings were transferred to complete darkness for 16 h before *Agrobacterium*-infiltration to increase the competency for transient transformation (Lee and Yang, 2006). We counted seedlings age from the day they were spread on Gamborg’s media to start germination.

### Agroinfiltration of *C. roseus* Seedlings

The transient expression assay utilized the non-tumorigenic *Agrobacterium tumefaciens* GV3101 (pMP90) strain (Koncz et al., 1992). *Agrobacteria* were transformed with expression plasmids by electroporation and plated on LB agar media with gentamicin (10 μg/ml) and appropriate antibiotics for selection of expression vectors. After verification by colony PCR, a single colony was cultivated in antibiotic-containing LB media overnight at 26°C and 250 RPM. Glycerol stocks were prepared from freshly growing liquid cultures and stored at −80°C for long-term storage.

Four days before agroinfiltration, *Agrobacteria* from the glycerol stocks were freshly streaked onto LB agar plates with gentamicin (10 μg/ml) and appropriate antibiotics and incubated 2–3 days at 26°C. A fresh single colony from the plate was used to inoculate 10 ml of LB liquid medium containing appropriate antibiotics and incubated overnight at 26°C at 250 RPM. To induce the virulence genes, *Agrobacteria* were centrifuged (7000×*g* for 6 min), resuspended in 10 ml of *Agrobacterium* minimal media with appropriate antibiotics and 100 μM acetosyringone (AS), and incubated for 3 h at 26°C and 250 RPM. The *Agrobacterium* minimal media consists of 5 g/L glucose, 1 g/L NH_4_Cl, 0.3 g/L MgSO_4_, 0.150 g/L KCl, 0.010 g/L CaCl_2_, 0.0025 g/L FeSO_4_, 3 g/L K_2_HPO_4_, 1 g/L NaH_2_PO_4_, and 0.195 g/L MES, buffered at pH = 5.5 (*Agrobacterium* Minimal Medium, bioWORLD).

In preparation for agroinfiltration, *Agrobacteria* cells were centrifuged (7000×*g* for 6 min) and resuspended to the desired OD at 600 nm in infiltration medium (Lee and Yang, 2006; 10 mM MgSO_4_, 10 mM MES, pH 5.6) freshly supplemented with 200 μM AS. Agroinfiltration was carried out with 10, 14, and 21-day old seedlings using either a needless syringe or under vacuum. For vacuum infiltration, 0.01% Silwet^®^ L-77 was added to the infiltration media.

#### Syringe Infiltration

A needleless 5 ml syringe was used to infiltrate *Agrobacterium* into the abaxial side of individual cotyledons in place. This method could not be used with 10-day-old seedlings as the cotyledons were smaller than the syringe. After syringe infiltration, seedlings were kept in Magenta boxes containing Gamborg’s media and maintained in the dark for 48 h and then transferred back to a 16 h/8 h photoperiod for 24 h.

#### Vacuum Infiltration

Seedlings (25–30) were gently transferred from the Magenta box to a 100 ml glass bottle containing 50 ml of infiltration media. Uncapped bottles were placed inside a vacuum desiccator, and vacuum was applied twice for 4 min, with an interval of no vacuum for 4 min. After vacuum infiltration, seedlings were transferred to Magenta boxes containing fresh Gamborg’s media, gently planting the roots into the media to help seedlings stand up. Seedlings were maintained in the dark for 48 h and then transferred to a 16 h/8 h photoperiod for 24 h (typically) or the desired time.

### *C. roseus* Developmental Stages

We evaluated the *Agrobacterium*-mediated transformation of *C. roseus* seedlings at three early developmental stages to minimize the time required to conduct the transient expression protocol. The developmental stages consisted of the following: (1) 10-day-old seedlings (7 days in the dark followed by 3 days in 16 h/8 h photoperiod) with small, open, and green cotyledons, (2) 14-day-old seedlings (7 days in the dark followed by 7 days in 16 h/8 h photoperiod) with bigger cotyledons but no visible true leaves, and (3) 21-day-old seedlings (7 days in the dark followed by 14 days in 16 h/8 h photoperiod) with the first set of true leaves at approximately 4 mm in length.

### Transient Transformation Evaluation

We evaluated the *Agrobacterium*-mediated transformation of *C. roseus* seedlings both qualitatively using the *GUS* gene and quantitatively using the luciferase gene. For qualitative evaluation, seedlings were transiently transformed with a plasmid encoding an intron-containing *β-glucuronidas*e gene (*GUS*) under the control of the cauliflower mosaic virus 2X 35S promoter (*CaMV2X35S*, Supplementary Figure S1 and Figure 6) and visualized with GUS staining.

**FIGURE 1:**
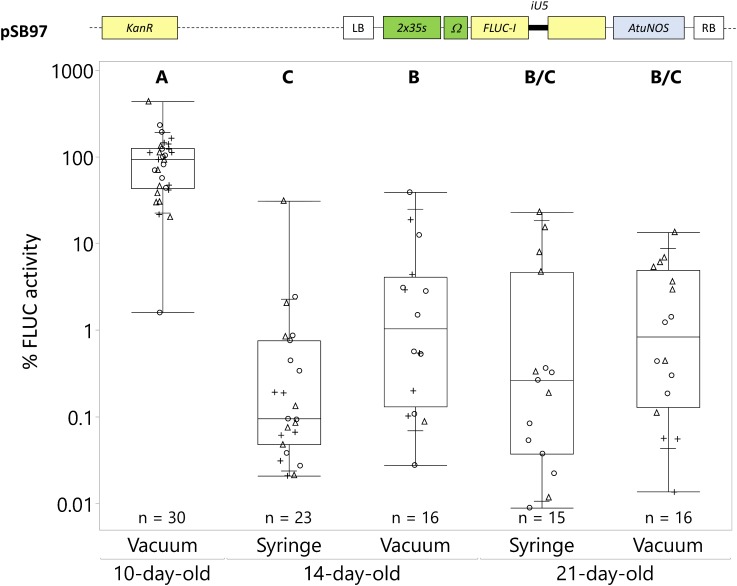
*Catharanthus roseus* seedling age and infection method significantly affect transient transformation efficiency. *C. roseus* seedlings at 10, 14, and 21-day-old were infiltrated with *A. tumefaciens* GV3101 (pMP90) containing the *FLUC-I* reporter (plasmid pSB97) at OD_600_ = 0.2, using either syringe or vacuum infiltration. Samples were taken at 3 dpi. FLUC activity was normalized to protein content. The average FLUC activity to protein content of 10-day-old vacuum infiltrated seedlings was used as reference (set to 100%) to determine the relative FLUC activity of each sample. The experiment was carried out in three independent assays (represented by the +, ○, Δ symbols). The box plot horizontal line shows the median, ends of the boxes show interquartile range, small marks show the 10th and 90th quantile and whiskers show lowest/highest data point. Different letters indicate values that are significantly different (Tukey-Kramer HSD test on log-transformed data, *p* < 0.05).

For quantitative evaluation, we measured the bioluminescence of seedlings transformed with the intron-containing firefly luciferase gene (*FLUC-I*) under the control of different promoters and the intron-containing *Renilla* luciferase gene (*RLUC-I*) under the control of the *Agrobacterium tumefaciens* promoter (*AtuMAS*, Figure 1). The FLUC signal was either normalized to the RLUC signal or protein content as quantified by the Bradford protein assay (Bio-Rad). For each experiment, one condition served as reference and its average normalized FLUC activity was set to 100%. To calculate the relative % activity, the normalized FLUC activity of each sample was divided by the average normalized FLUC activity of the reference. Calculating the relative % activity facilitated the statistical analysis of pooled data from three independent assays. For each condition of the assay, 10 biological repeats were conducted (if not otherwise specified); each biological repeat consisted of two 10-day-old seedlings or a single 17- and 24-day old seedlings. Statistical analysis of experimental data was carried out using JMP^®^, Version 13 (SAS Institute Inc., Cary, NC, United States, 1989–2007).

#### GUS Staining and Imaging

Plant tissue was stained with 5-bromo-4-chloro-3-indolyl glucuronide (X-Gluc) by incubating tissue in GUS staining solution (50 mM Na_3_PO_4_ buffer at pH 7.2, 1.0 mM EDTA, 0.1 mM K_3_Fe(CN)_6_, 0.1 mM K_4_Fe(CN)_6_, 20% (v/v) methanol, 700 μg/ml X-Gluc) at 25°C for 24 h. Plant tissue was then washed in 70% EtOH until the chlorophyll was completely removed. Images were acquired with a Nikon SMZ800 microscope (Nikon, Tokyo, Japan) and SPOT Insight CCD 2.0 Mp Color camera (Diagnostic Instruments, Inc., Sterling Heights, MI, United States).

#### Protein Extraction

Transformed seedlings were harvested, flash-frozen in liquid nitrogen, and kept at −80°C until protein extraction. Frozen tissue was pulverized using 3 mm beads in a bead beater at 4 m/s for 20 s (MP Biomedicals), transferred to ice, and 130 μl of extraction buffer was added (50 mM Na_3_PO_4_ at pH7.2, 1.0 mM EDTA) with 10 mM β-mercaptoethanol and 0.1% Triton-X 100 added fresh. After vortexing and centrifugation (21,000×*g* for 2 min), approximately 110 μl of supernatant was transferred to a 96-well plate and maintained on ice.

#### Firefly and *Renilla* Luciferase Assay

FLUC activity was analyzed using the Luc-Pair^TM^ Firefly Luciferase HT Assay while both FLUC and RLUC activity was analyzed using the Luc-Pair^TM^ Duo-Luciferase HT Assay Kit (Genecopeia). The plant protein extract (20 μl) was mixed with 20 μl of the appropriate reaction solution from the corresponding kit at room temperature. Samples were dispensed in every other well of a 96-well white plates (Corning^TM^ 3992) to avoid cross-talk between wells. Luminescence was read with a plate reader for 2 s to acquire relative light units (RLU).

#### Protein Quantification

The Bradford assay (BioRad) was used to quantify protein content. A protein standard curve from 62.5 to 3000 ng/μl of bovine serum albumin was prepared and a second order polynomial equation was used to fit the data. Samples were diluted 1:2 to ensure the values were within range of the calibration curve.

### Vector Construction

During vector construction, all PCR reactions required using Phusion High-Fidelity DNA Polymerase (New England BioLabs) to avoid introducing errors during PCR, and all primers used in this study are listed in Supplementary Table S1.

#### Vector Construction With Golden Gate Based Modular Cloning (MoClo)

All final vectors were constructed using Golden Gate based modular cloning (MoClo, Weber et al., 2011). MoClo uses standardized genetic elements (parts in L0 vectors) to assemble these into transcriptional units (L1 vectors). Multiple transcriptional units from L1 vectors can then be assembled in the final L2 vectors. Backbone vectors and Level 0 parts were taken from the Golden Gate MoClo Plant Parts Kit and Golden Gate MoClo Plant Toolkit (Weber et al., 2011; Engler et al., 2014) if not otherwise specified. Higher level vectors were assembled as described in Weber et al. (2011) to obtain the final vectors needed in this study.

The construction of novel L0 parts is described in Supplementary Materials (Methods). All new promoters and coding sequences were cloned into the L0 acceptor vectors as described in Weber et al. (2011). All newly constructed L0 MoClo vectors and final L2 vectors are deposited at Addgene (Addgene IDs 123180-123200).

The HcPro sequence (GB0067) was a gift from Dr. Diego Orzaez (Addgene plasmid # 68196, Sarrion-Perdigones et al., 2013). The vector is MoClo compatible.

#### Construction of a *VirGN54D* Containing L2 Acceptor Vector

pAGM4723 (Weber et al., 2011) was modified by inserting the *VirGN54D* gene using NEBuilder (New England BioLabs). An 1153 bp fragment containing the *VirGN54D* gene was amplified from pAD1289 (Hansen et al., 1994) with primers 1 and 2. The linearized pAGM4723 vector was obtained by amplification with primers 3 and 4. The *VirGN54D* fragment and linearized vector were assembled as described in the NEBuilder manual. The constructed vector, containing *VirGN54D* in the backbone, was named pSB90 – pAGM4723_VirGN54D and was deposited at Addgene (Addgene ID 123187).

## Results

### Optimum Transgene Expression Occurs With Vacuum Infiltration of Young Seedlings

In preliminary experiments, we assessed the amenability of *C. roseus* leaves to *Agrobacterium*-mediated transformation and observed high transformation with seedlings but decreased transformation with increasing leaf age (data not shown). Since seedlings exhibited the highest reporter gene expression and could be produced in a short period of time, we tested both syringe and vacuum infiltration on young seedlings (10, 14, and 21-day-old). Cotyledons of 10-day-old seedlings were too small to carry out syringe infiltration; therefore only the effect of vacuum infiltration was evaluated at this seedling age.

The highest transformation efficiency occurred in 10-day-old seedlings using vacuum infiltration, resulting in an 18–57 fold higher FLUC activity compared to any other condition (Figure 1). Both transformation methods produced significantly lower and less efficient transformations in 14 and 21-day-old seedlings. The transformation efficiency was not statistically different between vacuum infiltration and syringe infiltration in 14 and 21-day-old seedlings. Overall, vacuum infiltration of 10-day-old seedlings consistently resulted in high transformation efficiency with minimal tissue damage, unlike syringe infiltration. Also, vacuum infiltration required less tissue handling. Therefore, vacuum infiltration of 10-day-old seedlings was used for the optimization of other factors involved in the transient transformation process.

### High Transgene Expression Occurs at *Agrobacterium* Density of OD_600_ = 0.2 and 0.4

*Agrobacterium*-mediated transient transformation protocols in diverse plant species use bacterial density (OD_600_) in the infection medium from 0.01 to 2 (Wroblewski et al., 2005; Cazzonelli and Velten, 2006; Marion et al., 2008; Hosein et al., 2012). While low *Agrobacterium* densities yield low transgene expression, high densities (>OD_600_ = 1.0) can result in lower efficiency (Hosein et al., 2012), tissue yellowing, or wilting (Wroblewski et al., 2005). An optimum bacterial density yields higher transgene expression (Wroblewski et al., 2005; Marion et al., 2008) without tissue damage. Therefore, we evaluated the transient transformation efficiency of 10-day-old *C. roseus* seedlings using OD_600_ = 0.02, 0.2, and 0.4 during vacuum infiltration.

**FIGURE 2:**
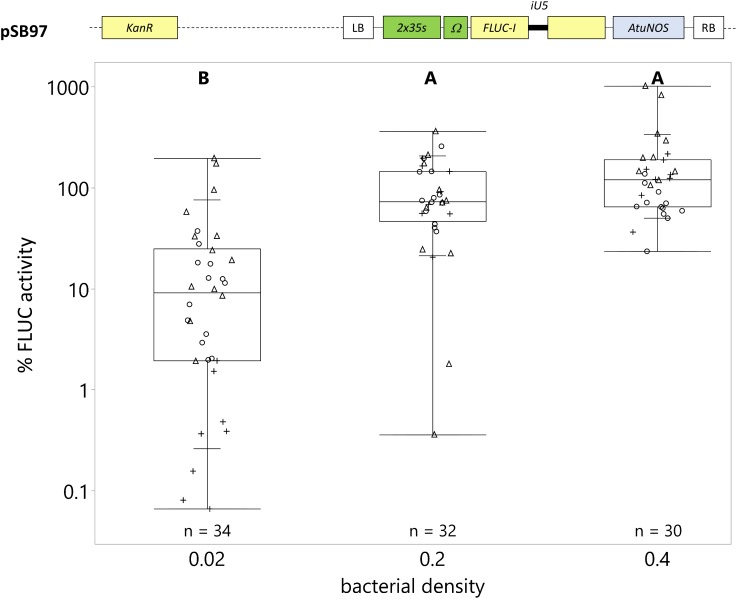
Transgene expression is directly correlated with bacterial density during vacuum infiltration. *C. roseus* seedlings (10-day-old) were vacuum infiltrated with *A. tumefaciens* containing the *FLUC-I* reporter (plasmid pSB97) at OD_600_ = 0.02, 0.2, and 0.4. Samples were taken at 3 dpi. FLUC activity was normalized to protein content. The average FLUC activity to protein content of seedlings infected at OD_600_ = 0.2 was used as reference (set to 100%) to determine the relative FLUC activity of each sample. The experiment was carried out in three independent assays (represented by the +, ○, Δ symbols). The box plot horizontal line shows the median, ends of the boxes show interquartile range, small marks show the 10th and 90th quantile and whiskers show lowest/highest data point. Different letters indicate values that are significantly different (Tukey-Kramer HSD test on log-transformed data, *p* < 0.05).

We found that the bacterial density had a significant effect on *C. roseus* transformation efficiency (Figure 2). Transformation efficiency increased directly with bacterial density. The average transformation efficiency at OD_600_ = 0.02, 0.2 (reference), and 0.4 was 24, 100, and 177%, respectively (Figure 2). Even though OD_600_ = 0.4 yielded the highest transformation efficiency with little tissue damage, we used OD_600_ = 0.2 in subsequent vacuum infiltrations to accommodate the combination of two *A. tumefaciens* strains carrying effector and reporter plasmids (see section “Dual-Luciferase Reporter System Is the Preferred Normalization Strategy for Reducing Inter-Experimental Variability” and “Transactivation of the *C. roseus* STR1 Promoter With ORCA3 and ZCT1”), which in total resulted in the highest bacterial concentration tested, OD_600_ = 0.4.

### Optimum Transgene Expression Occurs 3 Days Post-infection

Transgene expression is commonly evaluated 2–4 days post-infection (dpi) with *Agrobacterium* (Sparkes et al., 2006; Marion et al., 2008; Wu et al., 2014; Lu et al., 2017). Longer cultivation periods after infection may favor the accumulation of proteins or metabolites of interest. Therefore, we evaluated FLUC activity in seedlings 1, 2, 3, 6, and 9 dpi to obtain a time course of expression.

**FIGURE 3:**
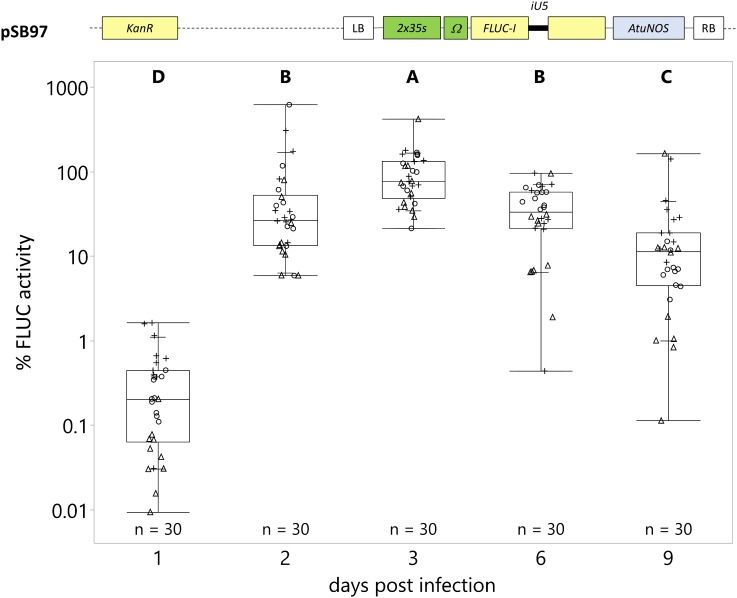
Strongest transgene expression is detected in seedlings 3 days post-infection (dpi). *C. roseus* seedlings (10-days-old) were vacuum infiltrated with *A. tumefaciens* containing the *FLUC-I* reporter (plasmid pSB97) at OD_600_ = 0.2. Samples were taken 1, 2, 3, 6, and 9 dpi. FLUC activity was normalized to protein content. The average FLUC activity to protein content of samples collected at 3 dpi was used as reference (set to 100%) to determine the relative FLUC activity of each sample. The experiment was carried out in three independent assays (represented by the +, ○, Δ symbols). The box plot horizontal line shows the median, ends of the boxes show interquartile range, small marks show the 10th and 90th quantile and whiskers show lowest/highest data point. Different letters indicate values that are significantly different (Tukey-Kramer HSD test on log-transformed data, *p* < 0.05).

The strongest FLUC activity was detected 3 dpi (Figure 3). At 1 dpi, FLUC activity was detectable at low levels, at 0.35% of the average FLUC activity at 3 dpi (reference, 100%). The average FLUC activity increased at 2 and 3 dpi (65 and 100%, respectively). At 6 and 9 dpi, the FLUC activity decreased to 40 and 21%, respectively. Therefore, we evaluated all further experiments at 3 dpi, unless otherwise noted.

### Constitutive Expression of Virulence Genes Enhances Transgene Expression

In *Agrobacterium*, the virulence (*Vir*) region of the Ti-plasmid encodes genes essential for DNA transfer to the plant. Transcription of the *Vir* genes is regulated by VirA and VirG in response to phenolic inducer(s) produced by plants such as acetosyringone (AS). Specifically, phosphorylation of VirG by VirA results in the transcription of *Vir* genes. In the mutated *VirGN54D*, an asparagine is substituted to aspartic acid at position 54, resulting in the inducer-independent activity of VirG (Pazour et al., 1992). Generally, AS is included during the pre-induction and infection steps to drive the expression of the *Vir* genes. In this paper, the *A. tumefaciens* strain used (GV3101) contains the pMP90 plasmid, which encodes all the native virulence genes. In this experiment, we evaluated whether the presence of *VirGN54D* could either replace AS or work synergistically with AS to improve transient transformation efficiency. The *VirGN54D* was either encoded on the same plasmid (pSB96) or on a different plasmid as the reporter gene (pSB97 + pAD1289) and electroporated into the GV3101 (pMP90) strain.

**FIGURE 4:**
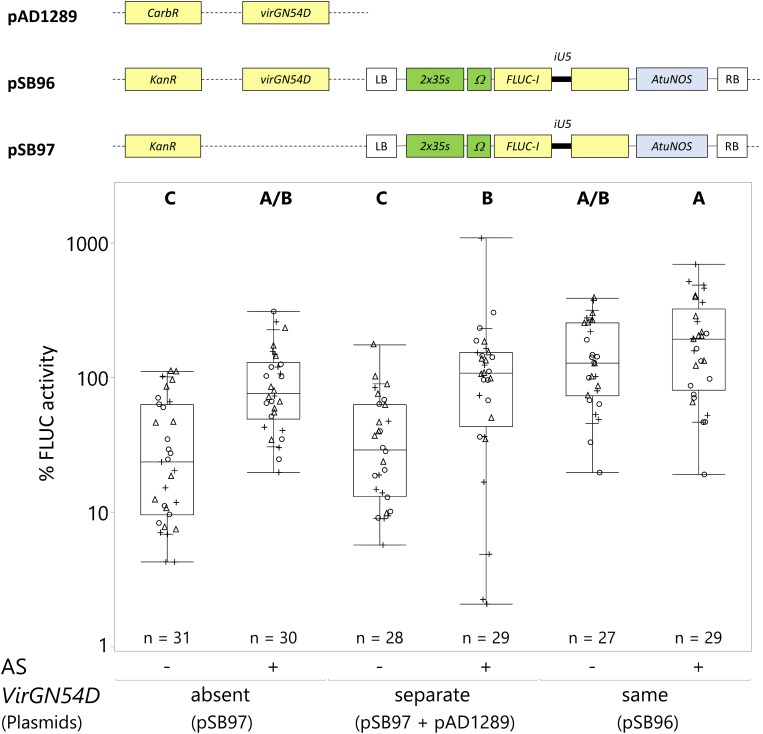
*VirGN54D* and acetosyringone synergistically improve *C. roseus* transient transformation. *C. roseus* seedlings (10-days-old) were vacuum infiltrated with one strain of *A. tumefaciens* at OD_600_ = 0.2, without (–) or with (+) AS. The *A. tumefaciens* strain contained either the reporter gene (pSB97) with no *VirGN54D* gene or with *VirGN54D* gene on a separate plasmid (pSB97+pAD1289), or with *VirGN54D* on the same plasmid as the *FLUC-I* reporter (pSB96). Samples were taken at 3 dpi. FLUC activity was normalized to protein content. The average FLUC activity to protein content of seedlings treated with AS but in the absence of *VirGN54D* was used as reference (set to 100%) to determine the relative FLUC activity of each sample. The experiment was carried out in three independent assays (represented by the +, ○, Δ symbols). The box plot horizontal line shows the median, ends of the boxes show interquartile range, small marks show the 10th and 90th quantile and whiskers show lowest/highest data point. Different letters indicate values that are significantly different (Tukey-Kramer HSD test on log-transformed data, *p* < 0.05).

Both *VirGN54D* and AS significantly increased *C. roseus* transformation efficiency (Figure 4). The addition of AS increased transformation efficiency when *VirGN54D* was either absent or on separate plasmids (absent and −AS = transformation efficiency of 37%; absent and +AS = 100% (reference); separate and −AS = 42%; separate and +AS = 141%; Figure 4). When encoded on the same plasmid as the reporter gene, *VirGN54D* with AS achieved the highest transformation efficiency (same and +AS = 220%; Figure 4). Therefore, we used *VirGN54D* in the backbone of the same plasmid as the reporter gene combined with AS addition in subsequent optimization experiments.

### Silencing Suppressors EnhanceTransgene Expression With ProlongedCultivation Post-infection

Silencing suppressors can disrupt the plant’s innate RNA silencing mechanisms and can therefore increase transgene expression levels over time in transient transformations (Pumplin and Voinnet, 2013). We evaluated the effect of the following three silencing suppressors on *C. roseus* transient transformation efficiency. The *Tobacco etch potyvirus* (TEV) helper-component proteinase (HcPro) was one of the first silencing suppressors discovered in plants (Anandalakshmi et al., 1998; Brigneti et al., 1998; Kasschau and Carrington, 1998). HcPro interferes with transgene-induced or virus-induced RNA silencing at the posttranscriptional level via modes that are not completely understood (reviewed in Voinnet, 2001). The *Tomato bushy stunt virus* (TBSV) suppressor of gene silencing, P19 (Silhavy et al., 2002), binds to small 21-nt RNAs, preventing their activity in AGO proteins and reducing the AGO1 protein accumulation through induction of miR168 (Várallyay et al., 2010). The V2 from *Tomato yellow leaf curl virus* (TYLCV) prevents new double-strand RNA synthesis by binding the RNA-DEPENDENT RNA POLYMERASE 6 (RDR6) cofactor SUPPRESSOR OF GENE SILENCING 3 (SGS3) (Zrachya et al., 2007; Glick et al., 2008).

**FIGURE 5:**
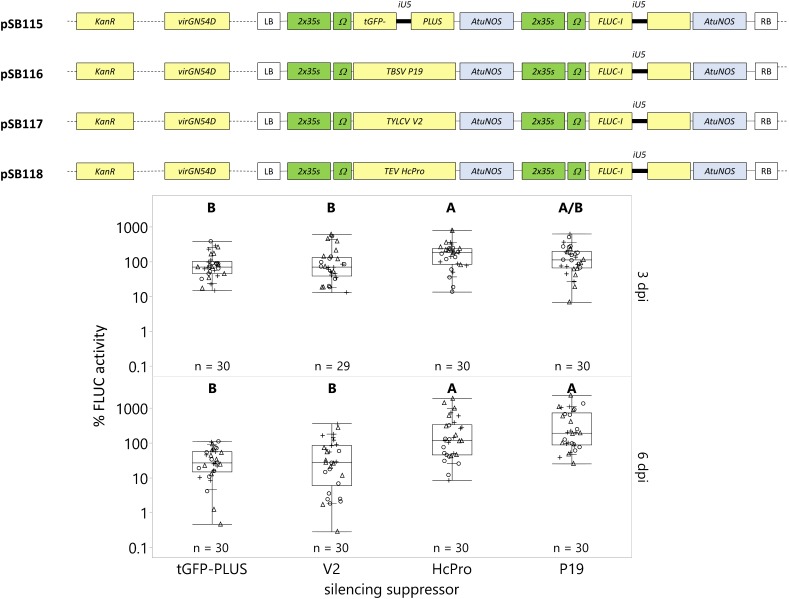
The silencing suppressors HcPro and P19 enhance transgene expression with prolonged cultivation post-infection. *C. roseus* seedlings (10-days-old) were vacuum infiltrated with *A. tumefaciens* containing the *FLUC-I* reporter and silencing suppressor within the same plasmid (TYLCV V2 – pSB117, TEV HcPro – pSB118, TBSV P19 – pSB116) or control (tGFP-PLUS – pSB115) at OD_600_ = 0.2. Samples were taken at 3 and 6 dpi. FLUC activity was normalized to protein content. The average FLUC activity to protein content of control samples (tGFP-PLUS) collected 3 dpi was used as reference (set to 100%) to determine the relative FLUC activity of each sample. The experiment was carried out in three independent assays (represented by the +, ○, Δ symbols). The box plot horizontal line shows the median, ends of the boxes show interquartile range, small marks show the 10th and 90th quantile, and whiskers show lowest/highest data point. Different letters indicate significantly different values within the 3 or 6 dpi groups (Tukey-Kramer HSD test on each group, on log-transformed data, *p* < 0.05).

In this study, we compared the effect of these silencing suppressors on transgene expression under *Agrobacterium*-mediated transient transformation after 3 dpi (optimum from section “Optimum Transgene Expression Occurs 3 Days Post-infection”) and 6 dpi, when the plant’s RNA silencing process is activated. Expression of the *FLUC-I* reporter gene in the presence of silencing suppressors (V2, HcPro, and P19) was compared against a no silencing control (tGFP-PLUS). At 3 dpi, only HcPro significantly increased FLUC activity (212%) relative to tGFP-PLUS (100%, Figure 5). At 6 dpi, transgene expression decreased in the absence of the silencing suppressors. For instance, the FLUC activity in the tGFP-PLUS control at 6 dpi was 38% of its expression at 3 dpi (consistent with our results in section “Optimum Transgene Expression Occurs 3 Days Post-infection”). HcPro and P19 strongly and significantly increased FLUC activity at 6 dpi. For instance, the FLUC activity was 8–11 fold higher compared to the tGFP-PLUS control at 6 dpi (HcPro = 310%; P19 = 446%). The silencing suppressor V2 did not significantly increase transgene expression at 3 or 6 dpi in our experiments and seems less suitable in the *C. roseus* system. Therefore, we recommend including HcPro and P19 silencing suppressor for applications requiring prolonged cultivation (6 dpi) post *Agrobacterium* infiltration.

### Transformation of *C. roseus* Seedlings Leads to an Even Histochemical GUS Staining of the Cotyledons With Minimal Tissue Damage

From our studies using luciferase reporters, we obtained the highest transformation efficiency using the following conditions: (1) 10-day-old seedlings transformed with *A. tumefaciens* GV3101 (pMP90) strain at OD_600_ = 0.2 using vacuum infiltration and harvested 3 dpi, and (2) the *A. tumefaciens* strain transformed with the intron-containing reporter gene and the *VirGN54D* within the same plasmid synergistically activated with AS.

**FIGURE 6:**
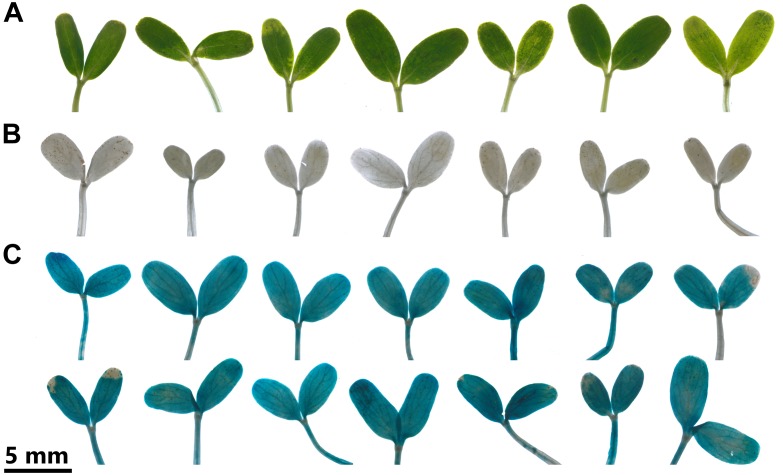
*Catharanthus roseus* seedlings after transformation with *A. tumefaciens*. *C. roseus* seedlings (10-day-old) were vacuum infiltrated with *A. tumefaciens* lacking the vector encoding the *GUS* reporter at OD_600_ = 0.4 **(A,B)** or containing the vector encoding the *GUS* reporter at OD_600_ = 0.2 (pSB161, C). Samples were taken at 3 dpi. Seedlings **(B,C)** underwent histochemical GUS assay and then were destained in 70% ethanol.

To determine whether EASI results in even transformation efficiency across seedling leaves, we transformed seedlings with the reporter *β-glucuronidase* (*GUS*), which enzymatically cleaves X-Gluc to produce a blue precipitate. This protocol resulted in minimal tissue damage at the harvest time point (3 dpi, Figure 6A). Endogenous β-glucuronidase-like (GUS-like) activity can be detected in specific tissues of various plant species (Hu et al., 1990); however, we detected no GUS-like activity in *C. roseus* seedlings after transformation with *A. tumefaciens* lacking a *GUS* reporter (Figure 6B). Transformation of seedlings with *A. tumefaciens* containing the *CaMV2x35s* driven *GUS* reporter at OD_600_ = 0.2 led to an even transformation of the cotyledons (Figure 6C). Hence, our EASI protocol promotes homogenous and high expression of the transgene in the cotyledons of *C. roseus* seedlings.

### Dual-Luciferase Reporter System Is the Preferred Normalization Strategy for Reducing Inter-Experimental Variability

For accurate measurement of expression in promoter transactivation or promoter activity studies, a sensitive reporter gene with a wide dynamic range and rapid readout is desired. Luciferase assays provide high sensitivity and time-resolution for measuring promoter activity. While the *GUS* reporter is a valuable reporter for visualizing the evenness of expression across the tissue (Ruijter et al., 2003; Koo et al., 2007; Velten et al., 2008), *GUS* has a longer mRNA and protein half-life, which can bias the interpretation of promoter activity (Ruijter et al., 2003). Therefore, two luciferases (firefly luciferase and *Renilla* luciferase) were selected for quantifying promoter activity and normalizing transformation efficiency.

**FIGURE 7:**
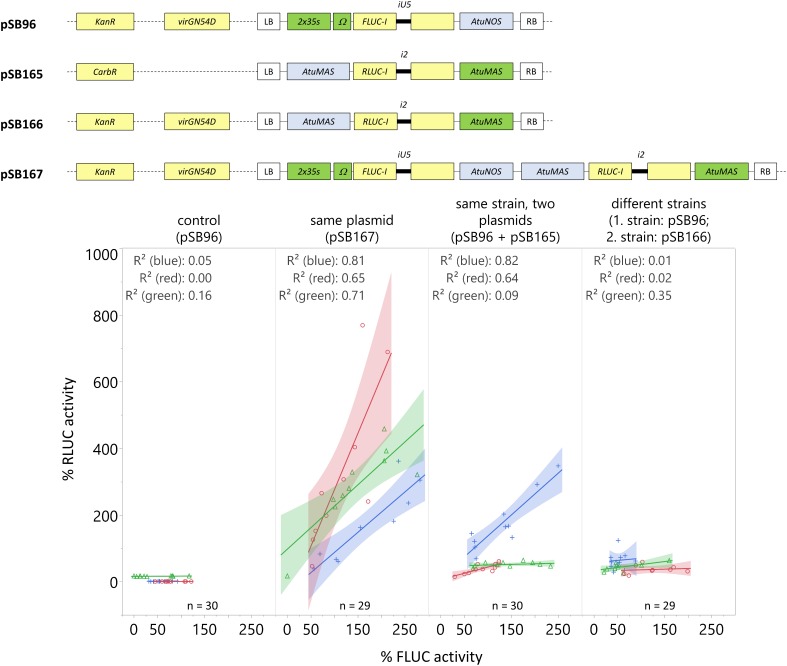
Including the normalization reporter *RLUC-I* on the same plasmid as the reporter *FLUC-I* provides consistently high RLUC signal, and is highly correlated with FLUC signal. *C. roseus* seedlings (10-day-old) were vacuum infiltrated with *A. tumefaciens* containing: (I) the *FLUC-I* reporter (Negative control – pSB96), (II) the *FLUC-I* reporter and the *RLUC-I* reporter within the same plasmid (Same plasmid – pSB167), (III) the *FLUC-I* reporter and the *RLUC-I* reporter in different plasmids but same strain of *A. tumefaciens* (Same strain, Two plasmids – pSB96, pSB165), (IV) the *FLUC-I* reporter and the RLUC-I reporter in different plasmids and different strains of *A. tumefaciens*, co-infiltrated into seedlings (Different strains – pSB96, pSB166) at OD_600_ = 0.2 for each strain. Samples were taken 3 dpi. The experiment was carried out in three independent assays (represented by blue, red, and green lines). For each independent assay, the FLUC activity of each sample was normalized to the average FLUC signal, and the RLUC activity of each sample was normalized to the average RLUC signal. Linear fit and corresponding R-squared values for the correlation between FLUC and RLUC signal are shown for each condition, in each experiment. The shaded region represents the 95% confidence interval of the linear fit.

Normalization of FLUC signal to protein content has been used to account for variations in the amount of tissue (Ow et al., 1986). To account for variations in transformation efficiency across seedlings within an experiment, the activity of *Renilla* luciferase (RLUC) is generally used to normalize the FLUC reporter signal. Previous *Agrobacterium*-mediated transient assays normalized the transformation signal in the following ways: the reporter and normalization signal either (1) on the same plasmid (Liu et al., 2014; Moyle et al., 2017), (2) on different plasmids transformed into the same *Agrobacterium* strain (Cazzonelli and Velten, 2006), or (3) on different plasmids in different *Agrobacterium* strains and co-infiltrated into the seedlings (Van Moerkercke et al., 2011). To determine the optimal method for normalizing transformation efficiencies, we expressed *RLUC-I* and *FLUC-I* under the control of constitutive promoters (Figure 7) using these three approaches and determined the correlation between the two signals.

In Figure 7 (from left to right), the negative control, containing *FLUC-I* only (pSB96), showed no RLUC signal, confirming that the FLUC signal was properly quenched. Including the normalization gene (*RLUC-I*) on the same plasmid as the reporter gene (*FLUC-I*) consistently yielded a high signal and a high correlation between RLUC and FLUC for all replicate experiments (*R*^2^ from 0.65 to 0.81, Figure 7). Infiltrating seedlings with *A. tumefaciens* carrying the normalization and reporter genes on separate plasmids showed variable results. Co-infiltrating seedlings with two strains of *A. tumefaciens* carrying *FLUC-I* and *RLUC-I* separately resulted in minimal correlation between FLUC and RLUC signal.

Therefore, we recommend including the normalization gene (*RLUC-I*) on the same plasmid as the reporter gene (*FLUC-I*) to account for variations in transformation efficiency across seedlings within an experiment.

### Transactivation of the *C. roseus* STR1 Promoter With ORCA3 and ZCT1

Next, we applied EASI to study the transactivation of the *CroSTR1* promoter by a known activator (ORCA3, van der Fits and Memelink, 2000, 2001) and repressor (ZCT1, Pauw et al., 2004) in *C. roseus* seedlings. Seedlings were infiltrated with two *A. tumefaciens* strains (OD_600_ = 0.2 each or final OD_600_ = 0.4), one containing the reporter construct and the other containing the effector construct. The reporter construct consisted of the *CroSTR1* promoter driving *FLUC-I* and the *AtuNOS* promoter driving *RLUC-I* for normalization. The *CroSTR1* and *AtuNOS* promoters were placed in opposite orientations on the T-DNA, maximizing the distance between the promoters to avoid crosstalk between the promoters. The effector construct consisted of the *CaMV2x35s* promoter driving *GUS* (negative control), *ORCA3*, or *ZCT1*. An effector construct expressing *GUS* was used as the reference and GUS staining was used to evaluate transformation success.

**FIGURE 8:**
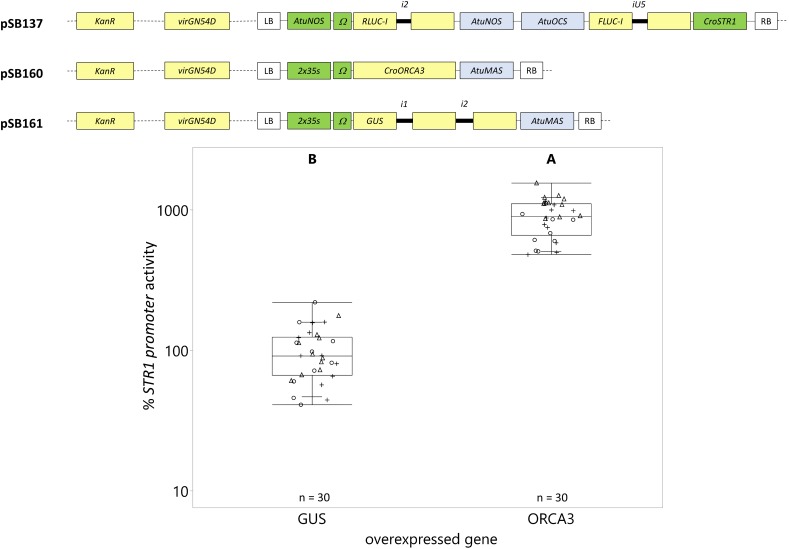
Transactivation of the **CroSTR1** promoter with ORCA3. **C. roseus** seedlings (10-days-old) were vacuum infiltrated with a combination of two strains of **A. tumefaciens** (1:1 volume ratio): (I) strain containing the **CroSTR1** promoter driven **FLUC-I** reporter and the **AtuNOS** promoter driven **RLUC-I** normalization reporter (plasmid pSB137) at OD_600_ = 0.2, and (II) strain containing a **CaMV2x35s** driven gene for transactivation (GUS as control – pSB161 or ORCA3 – pSB160) at OD_600_ = 0.2. Samples were taken 3 dpi. The **CroSTR1** promoter activity is the FLUC to RLUC activity ratio for each sample normalized to the FLUC to RLUC activity ratio of the GUS control (set to 100%). The experiment was carried out in three independent assays (represented by the +, ○, Δ symbols). The box plot horizontal line shows the median, ends of the boxes show interquartile range, small marks show the 10th and 90th quantile, and whiskers show lowest/highest data point. Different letters indicate significantly different values. **ORCA3** overexpression significantly increased the expression of the **STR1** promoter (Student’s **t**-test on log-transformed data, **p** < 0.0001).

**FIGURE 9:**
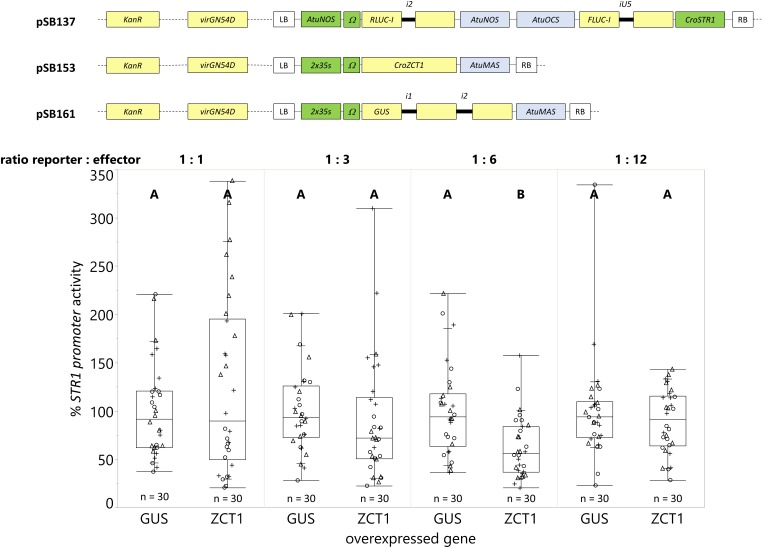
A ratio of 1-part reporter strain and 6-parts effector strain provides the highest sensitivity in transactivation assays. *C. roseus* seedlings (10-days-old) were vacuum infiltrated with a combination of two strains of *A. tumefaciens* (total OD_600_ = 0.4): (I) strain containing the *CroSTR1* promoter driven *FLUC-I* reporter and the *AtuNOS* promoter driven *RLUC-I* normalization reporter (plasmid pSB137), and (II) strain containing a *CaMV2x35s* driven effector for transactivation (GUS as control – pSB161 or ZCT1 – pSB153). Samples were taken 3 dpi. For each reporter to effector ratio, the *CroSTR1* promoter activity is the FLUC to RLUC activity ratio for each sample normalized to the FLUC to RLUC activity ratio of the GUS control (set to 100%). The experiment was carried out in three independent assays (represented by the +, ○, Δ symbols). The box plot horizontal line shows the median, ends of the boxes show interquartile range, small marks show the 10th and 90th quantile, and whiskers show lowest/highest data point. Different letters indicate significantly different values. *ZCT1* overexpression significantly decreased the expression of the *STR1* promoter in the 1:6 ratio, but not in the other ratios (Student’s *t*-test comparing each condition to its GUS control on log-transformed data, *p* < 0.05).

Overexpression of *ORCA3* led to a 9-fold increase in expression compared to the overexpression of the *GUS* control (Figure 8). However, overexpression of *ZCT1* did not repress the *CroSTR1* promoter activity (Figure 9). Therefore, the ratio of the strains was tested to optimize reporter signal and effector activity (1 part reporter strain to 3, 6, or 12 parts of effector strain while maintaining a final OD_600_ = 0.4). Supplementary Figure S2 shows a decrease in the activity of the normalization gene (*RLUC-I)* as the ratio of effector to reporter increases, as expected. Transactivation with ratios of 1:3, 1:6, and 1:12 led to a decrease of the *CroSTR1* promoter activity, but the activity of the *RLUC* was too low and unreliable at the 1:12 ratio and will be excluded in future studies (Supplementary Figure S2). The ratio that showed high reliable signal and significance was the 1:6 ratio. At the 1:6 ratio, the overexpression of *ZCT1* repressed the expression of the *CroSTR1* promoter by 38%. Therefore, EASI is an effective method for assessing both activation and repression of promoter activity in *C. roseus*.

## Discussion

In this paper, we present the development of an efficient *Agrobacterium*-mediated seedling infiltration (EASI) method for transformation of *C. roseus* seedlings, and demonstrate its applicability for the study of transcriptional regulation of TIA biosynthetic genes. This protocol can now be applied for the rapid assessment of the function of genes *in planta*, particularly for overexpressing transcription factor candidates and monitoring the expression of the genes involved in TIA biosynthesis. Our EASI method is complementary to viral induced gene silencing, which is used to silence the target gene (Liscombe and O’Connor, 2011). Previously, we developed an *Agrobacterium* co-cultivation method for transforming *C. roseus* leaf tissue (Weaver et al., 2014). Here, we have completely revised and markedly improved our previous transient expression method and increased transformation efficiency by (1) using vacuum infiltration, (2) introducing a constitutively active *VirG* gene, and (3) improving construct design, as detailed below.

### Significant Increases in Transformation Efficiency With Vacuum Infiltration of Young Seedlings

Of all the parameters tested, vacuum infiltration of young seedlings (10-day-old) resulted in the strongest increase in transgene expression, at 18–57 times higher than either vacuum or syringe infiltration of other seedling ages. Other parameters, including *Agrobacterium* density and time point of maximum transgene expression, increased transgene expression to a lesser extent, up to 100% increase (Figure 2, 3). Vacuum infiltration offers other advantages over syringe infiltration, including ease of scale-up, minimum handling, and minimal tissue damage compared to syringe infiltration of individual seedlings (Figure 1). Minimizing tissue damage is important because tissue damage could activate expression of stress responsive transcription factors and confound transactivation results.

### Increased Transformation Efficiency With Constitutively Active VirG and Silencing Suppressors

Incorporating the constitutively active *VirG* (*VirGN54D*, Pazour et al., 1992) also resulted in increased expression, particularly when the mutated *VirG* gene was encoded on the same plasmid as the reporter gene. It is possible including *VirGN54D* on the same plasmid as the reporter gene requires lower energy consumption or is less stressful for *Agrobacterium* to maintain (i.e., one plasmid with one antibiotic resistance gene) than on two separate plasmids (i.e., with two different antibiotic resistances). This construct (reporter and *VirGN54D* on the same plasmid) in combination with AS addition led to the highest transgene expression with a 120% increase above that of the reference condition (*VirGN54D* absent, + AS).

Commonly, transient transgene expression is high at 2–3 dpi but decreases with prolonged cultivation periods, as tissue dies due to the *Agrobacterium* infection or silencing of the transgene occurs. For example, Zrachya et al. (2007) transformed *Nicotiana benthamiana* leaves by agroinfiltration and observed high mRNA levels of the transgene (*GFP*) at 2 dpi but undetectable transgene expression at 7 dpi. The silencing suppressors V2, P19, and HcPro prolonged transgene expression in *Nicotiana benthamiana* (Zrachya et al., 2007). Similarly, we demonstrate that HcPro significantly increased transgene activity by 212% compared to the control at 3 dpi and up to 10-fold compared to the control at 6 dpi (Figure 5), while V2 is a less potent silencing suppressor in *C. roseus*.

### Construct Design Facilitates Both High Expression Level and Appropriate Normalization of Transformation Efficiency

When performing transient expression assays, the reporter gene activity needs to be normalized to account for potential differences in transformation efficiency and in amount of analyzed tissue. Normalization of reporter gene activity by protein content can account for variations in the amount of tissue, but not in transformation efficiency. Transforming two different *A. tumefaciens* strains with the respective reporter genes (e.g., firefly luciferase and *Renilla* luciferase) is the most convenient approach; no complex vector needs to be constructed and the plasmids can have the same origin of replication and antibiotic selection marker. Unfortunately, we found that both the overall signal and the correlation between the two reporter gene activities in this configuration were low (Figure 7). Another convenient approach is to transform one *A. tumefaciens* strain with two different vectors; the vectors should have different origins of replications with different incompatibly groups, and different antibiotic resistances to allow selection of colonies containing both vectors. Although good reporter gene expression and correlation was observed in the first experiment (Figure 7; *R*^2^ = 0.823), this trend was not consistent in subsequent replicate experiments (*R*^2^ = 0.644 and 0.090). Incorporating two plasmids within the same strain adds additional stress to the *Agrobacterium*, and may introduce variations in plasmid copy number or in T-DNA transfer rates.

In contrast, designing the construct with the reporter gene (*FLUC-I*) and the normalization gene (*RLUC-I*) on the same plasmid yielded a high signal and a high correlation between RLUC and FLUC (Figure 7). We therefore recommend the construction of one vector with both reporter genes, which has not always been a common practice. While cloning is more extensive, the construction of one vector with both reporter genes avoids the risk of plasmid instability associated with two vectors within the same strain. Furthermore, Gibson assembly (Gibson et al., 2009) or Golden Gate-based methods like MoClo (Weber et al., 2011) now provide easy to use and fast methods for the construction of complex vectors.

### Application of EASI for the Transactivation of the *C. roseus* STR1 Promoter With ORCA3 and ZCT1

We chose the well-studied transactivation and repression of the *CroSTR1* promoter by the activator ORCA3 and repressor ZCT1 to demonstrate the applicability of the EASI method. ORCA3 expression induced a 9-fold increase in the *CroSTR1* promoter activity (Figure 8). This is comparable to the previously reported transactivation of the *CroSTR1* promoter with ORCA3, which led to an ∼10- to 25-fold increase in tobacco protoplasts (Van Moerkercke et al., 2015; Paul et al., 2017) and a ∼2.5-fold increase in *N. benthamiana* leaves (Van Moerkercke et al., 2015). The *CroSTR1* promoter can be repressed by ZCT1 in *C. roseus* cells (Pauw et al., 2004). We did not observe any significant repression in *C. roseus* seedlings using a 1 to 1 ratio of the *Agrobacterium* strain containing the *CroSTR1* promoter driving reporter to the *Agrobacterium* strain containing the ZCT1 effector (Figure 9). A ratio of 1:6 provided maximum effect (38% decrease) from the effector strain while maintaining relatively high and reliable reporter gene expression. These results demonstrate the application of the EASI method to study novel transcriptional regulation of TIA biosynthesis in *C. roseus*.

## Conclusion

The described method achieves uniform and high transformation of the cotyledons and has the potential for many different applications in *C. roseus*, such as studying transcriptional regulation and protein localization. Therefore, EASI provides a timely tool for the community to rapidly assess the function of genes discovered through the availability of *C. roseus* transcriptome and genome resources. In addition, the vectors constructed in this study have been made available through Addgene to other researchers and can be applied for optimizing transient transformations and transactivation assays in other plant systems. These constructs include intron-containing reporter genes (for *Agrobacterium*-mediated transformation), the constitutively expressed virulence G gene, and the silencing suppressors, which together can result in highly efficient transient transformation.

## Author Contributions

SM, DB-F, EC, and CL-P conceived and designed the research. SM, DB-F, LC, and SS performed the experiments. SM, DB-F, LC, SS, EC, and CL-P analyzed the data. SM, DB-F, LC, EC, and CL-P wrote the manuscript.

## Conflict of Interest Statement

The authors declare that the research was conducted in the absence of any commercial or financial relationships that could be construed as a potential conflict of interest.

## Footnotes

1 http://medicinalplantgenomics.msu.edu

2 https://bioinformatics.tugraz.at/phytometasyn/

3 https://sites.google.com/a/ualberta.ca/onekp/

## Supplementary Material

FIGURE S1 Binary vectors used for *C. roseus* transformations. The plasmid pAD1289 was kindly provided by Prof. A. Das. All pSB vectors were constructed using MoClo (Weber et al., 2011; Engler et al., 2014). Level 1 vector parts for pSB96, pSB115-118, pSB137, pSB153, pSB160-161, and pSB166-167 were assembled in the newly constructed L2 acceptor vector pSB90 (see section “Materials and Methods” for detail). pSB97 and pSB153 were assembled in the pAGM4723 vector (Weber et al., 2011). Promoters including 5′UTRs are shown in green, coding sequences in yellow, and terminators in blue. Introns are indicated with a black bar within the coding sequence. Vectors and vector files of all pSB plasmids are deposited at Addgene (Addgene ID 123180–123200).

FIGURE S2 ZCT1 does not repress the *AtuNOS* promoter and a 1:12 ratio does not provide sufficient RLUC signal for normalization. *C. roseus* seedlings (10-days old) were vacuum infiltrated with a combination of two strains of *A. tumefaciens* (total OD_600_ = 0.4): (I) strain containing the *CroSTR1* promoter driven *FLUC-I* reporter and the *AtuNOS* promoter driven *RLUC-I* normalization reporter (plasmid pSB137), and (II) strain containing a *CaMV2x35s* driven effector for transactivation (GUS as control – pSB161 or ZCT1 – pSB153). Samples were taken 3 dpi. The experiment was carried out in three independent assays (represented by the +, ○, Δ symbols). The box plot horizontal line shows the median, ends of the boxes show interquartile range, small marks show the 10th and 90th quantile, and whiskers show lowest/highest data point. *ZCT1* overexpression did not significantly decrease the expression of the *AtuNOS* promoter (student’s *t*-test comparing each condition to its GUS control on log-transformed data, *p* < 0.05).

TABLE S1 Primers used for the construction of new vectors.

TABLE S2 Construction of level 0 vector parts.
