# Supplementary figures and images for: EASI Transformation: An Efficient Transient Expression Method for Analyzing Gene Function in Catharanthus roseus Seedlings
Source: Front Plant Sci. 2019 Jun 11;10:755. doi: 10.3389/fpls.2019.00755 (PMC6585625; doi:10.3389/fpls.2019.00755)

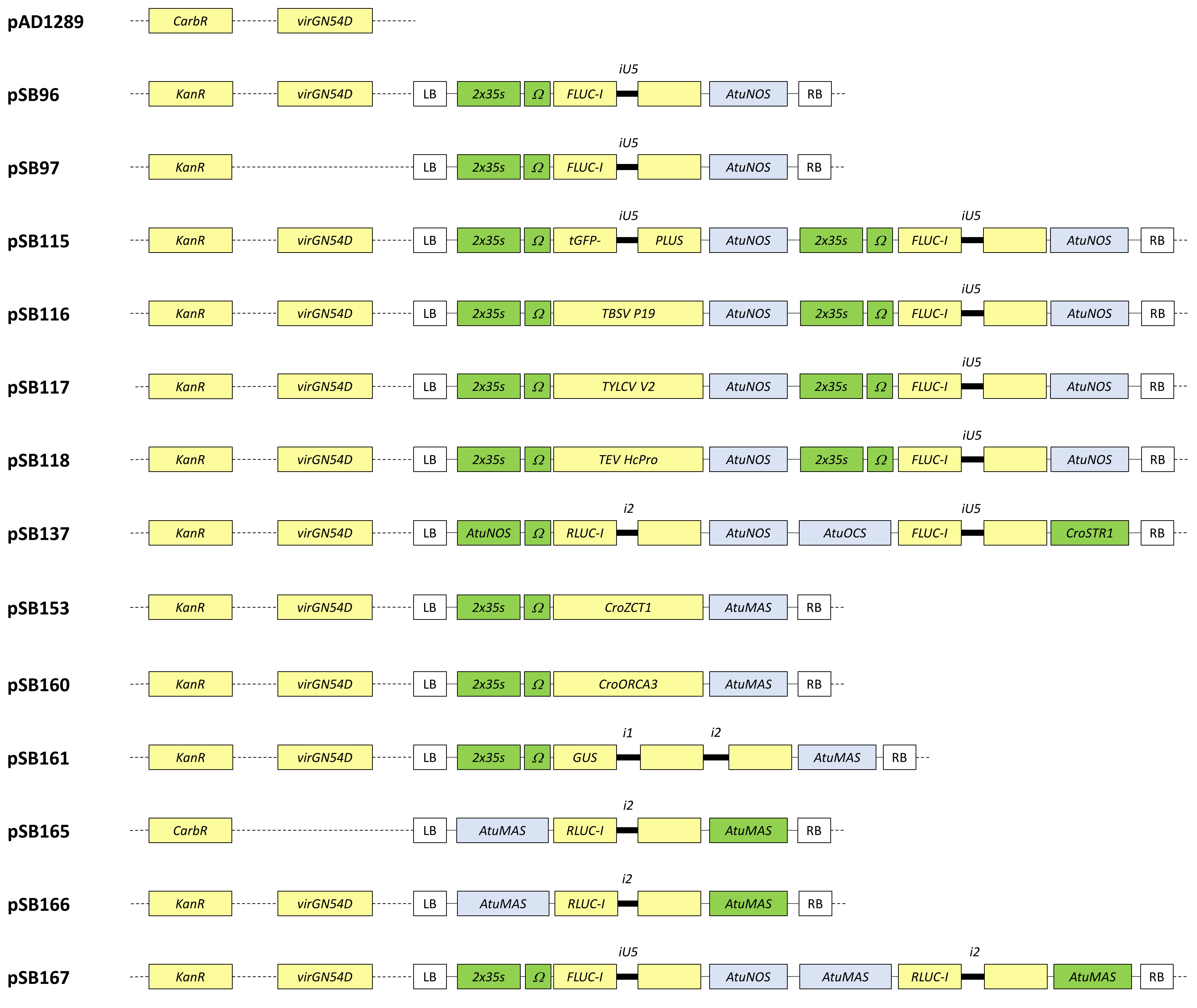

Supplement: FIGURE S1 — Binary vectors used for C. roseus transformations. The plasmid pAD1289 was kindly provided by Prof. A. Das. All pSB vectors were constructed using MoClo (Weber et al., 2011; Engler et al., 2014). Level 1 vector parts for pSB96, pSB115-118, pSB137, pSB153, pSB160-161, and pSB166-167 were assembled in the newly constructed L2 acceptor vector pSB90 (see section “Materials and Methods” for detail). pSB97 and pSB153 were assembled in the pAGM4723 vector (Weber et al., 2011). Promoters including 5′UTRs are shown in green, coding sequences in yellow, and terminators in blue. Introns are indicated with a black bar within the coding sequence. Vectors and vector files of all pSB plasmids are deposited at Addgene (Addgene ID 123180–123200). [file Image_1.TIF]

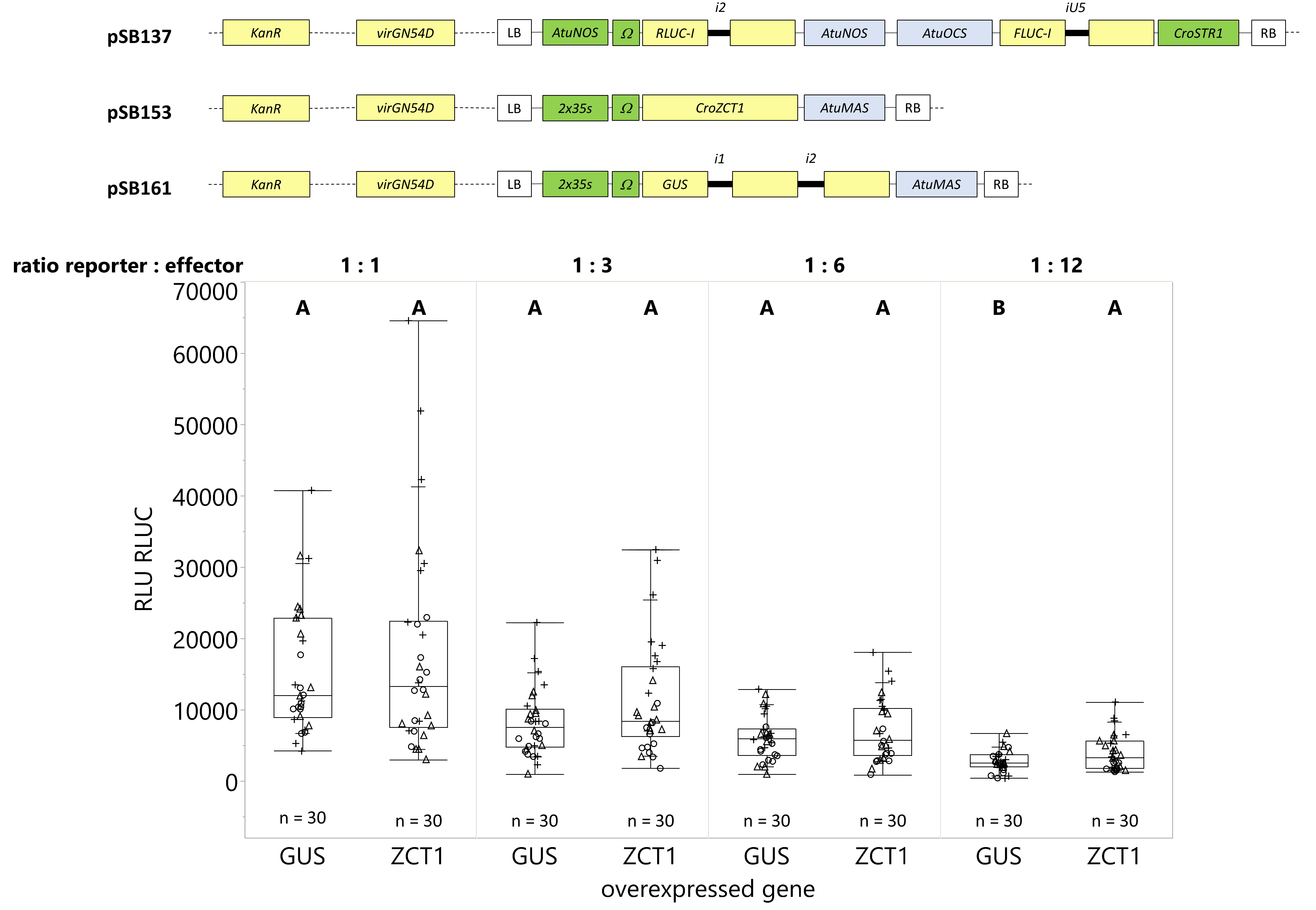

Supplement: FIGURE S2 — ZCT1 does not repress the AtuNOS promoter and a 1:12 ratio does not provide sufficient RLUC signal for normalization. C. roseus seedlings (10-days old) were vacuum infiltrated with a combination of two strains of A. tumefaciens (total OD600 = 0.4): (I) strain containing the CroSTR1 promoter driven FLUC-I reporter and the AtuNOS promoter driven RLUC-I normalization reporter (plasmid pSB137), and (II) strain containing a CaMV2x35s driven effector for transactivation (GUS as control – pSB161 or ZCT1 – pSB153). Samples were taken 3 dpi. The experiment was carried out in three independent assays (represented by the +, ○, Δ symbols). The box plot horizontal line shows the median, ends of the boxes show interquartile range, small marks show the 10th and 90th quantile, and whiskers show lowest/highest data point. ZCT1 overexpression did not significantly decrease the expression of the AtuNOS promoter (student’s t-test comparing each condition to its GUS control on log-transformed data, p < 0.05). [file Image_2.TIF]
