# Supplementary material for: EASI Transformation: An Efficient Transient Expression Method for Analyzing Gene Function in Catharanthus roseus Seedlings
Source: Front Plant Sci. 2019 Jun 11;10:755. doi: 10.3389/fpls.2019.00755 (PMC6585625; doi:10.3389/fpls.2019.00755)
Supplement: TABLE S2 — Construction of level 0 vector parts. [file Table_2.docx]

# *Supplementary Material*

# Supplementary Methods: Construction of level 0 vector parts

Firefly luciferase with intron (***FLUC-I***) was cloned by amplifying the *Photinus pyralis* luciferase coding sequence from pICSL80001 (Engler et al., 2014) into two fragments with primers 5 and 6, as well as primers 9 and 10. The U5 small nuclear ribonucleoprotein component (*A. thaliana*) intron was amplified from pICSL50016 (Engler et al., 2014) with primers 7 and 8. All three fragments were assembled in a MoClo *Bpi*I reaction into pICH41308 (Weber, Engler, Gruetzner, Werner, & Marillonnet, 2011), with the intron separating the coding sequences. The resulting vector was named pSB63 - pL0_fLUC-I (CDS1) (Addgene ID 123180).

A plant-codon optimized *Renilla* luciferase with intron (***RLUC-I***) was designed. First, *Renilla* luciferase from the plasmid pCMV-IRES-Renilla Luciferase-IRES-Gateway-Firefly Luciferase (pIRIGF; William Kaelin; Addgene plasmid # 101139; Lu et al., 2014) was codon optimized for plants using the “graphical codon usage analyser” (Fuhrmann, Hausherr, Ferbitz, Scho, & Hegemann, 2004). Then, the i2 intron, denoted as the second intron from pICH75111 (Engler et al., 2014), was inserted into the *RLUC* coding sequence between a potential splice site (AG|GT). Finally, a MoClo compatible gBlock containing the *RLUC-I* sequence was synthesized by IDT (INTEGRATED DNA TECHNOLOGIES) and cloned into the CDS1 acceptor vector pICH41308 (Weber et al., 2011). The resulting vector was named pSB123 - pL0_rLUC-I (CDS1) (Addgene ID 123181).

A MoClo compatible *Tomato yellow leaf curl virus* (*TYLCV*) ***V2*** silencing suppressor gBlock was synthesized by IDT (INTEGRATED DNA TECHNOLOGIES) based on NCBI Reference Sequence NC_004005.1 (Morilla et al., 2005) and cloned into pICH41308 (Weber et al., 2011). The resulting vector was named pSB61 - pL0_V2 (CDS1) (Addgene ID 123183).

The C-terminal tag ***tGFP-PLUS*** sequence from pICSL50016 (Engler et al., 2014) was amplified with primer 11 and 12 and inserted into the CDS1 acceptor vector pICH41308 (Weber et al., 2011) in a *Bpi*I MoClo reaction. The resulting vector was named pSB65 - pL0_tGFP-I (CDS1) (Addgene ID 123182).

The *C. roseus* ***ZCT1*** CDS (GenBank: AJ632082.1) was amplified from *C.* *roseus* cDNA with the primer 13 and 14 and was inserted into the CDS1 acceptor vector pICH41308 (Weber et al., 2011) in a *Bpi*I MoClo reaction. The resulting vector was named pSB139 - pL0_ZCT1 (CDS1) (Addgene ID 123184).

The *C. roseus* ***ORCA3*** CDS (GenBank: EU072424.1) was amplified from *C.* *roseus* cDNA with the primer 15 and 16, as well as primer 17 and 18 (replacing MoClo incompatible sites with synonymous codons) and were inserted into the CDS1 acceptor vector pICH41308 (Weber et al., 2011) in a *Bpi*I MoClo reaction. The resulting vector was named pSB142 - pL0_ORCA3 (CDS1) (Addgene ID 123185).

The *C.* *roseus* ***STR1*** promoter and 5’ UTR (GenBank: Y10182.1; Pasquali, Erven, Ouwerkerk, Menke, & Memelink, 1999) was amplified from *C.* *roseus* gDNA with primer 19 and 20 and was inserted into the Pro + 5U acceptor vector pICH41295 (Weber et al., 2011) in a *Bpi*I MoClo reaction. The resulting vector was named pSB62 - pL0_STR (pro + 5U) (Addgene ID 123186).

The newly constructed L0 vector parts and L0 vector parts from the Golden Gate MoClo Plant Parts Kit (Engler et al., 2014) were used to construct L1 vectors and the final pSB vectors (L2 vectors) as described in Weber et al., 2011.Vector cartoons of all final vectors used in this study can be found in Figure S1.

# References

Engler, C., Youles, M., Gruetzner, R., Ehnert, T. M., Werner, S., Jones, J. D. G., … Marillonnet, S. (2014). A Golden Gate modular cloning toolbox for plants. *ACS Synthetic Biology*, *3*(11), 839–843. https://doi.org/10.1021/sb4001504

Fuhrmann, M., Hausherr, A., Ferbitz, L., Scho, T., & Hegemann, P. (2004). Monitoring dynamic expression of nuclear genes in Chlamydomonas reinhardtii by using a synthetic luciferase reporter gene. *Plant Molecular Biology*, *55*(6), 869–881.

Lu, G., Middleton, R. E., Sun, H., Naniong, M. V., Ott, C. J., Mitsiades, C. S., … Kaelin, W. G. (2014). The myeloma drug lenalidomide promotes the cereblon-dependent destruction of ikaros proteins. *Science*, *343*(6168), 305–309. https://doi.org/10.1126/science.1244917

Morilla, G., Janssen, D., García-Andrés, S., Moriones, E., Cuadrado, I. M., & Bejarano, E. R. (2005). Pepper (Capsicum annuum) is a dead-end host for Tomato yellow leaf curl virus. *Phytopathology*, *95*(9), 1089–1097. https://doi.org/10.1094/PHYTO-95-1089

Pasquali, G., Erven, A. S. W., Ouwerkerk, P. B. F., Menke, F. L. H., & Memelink, J. (1999). The promoter of the strictosidine synthase gene from periwinkle confers elicitor-inducible expression in transgenic tobacco and binds nuclear factors GT-1 and GBF. *Plant Molecular Biology*, *39*(6), 1299–1310. https://doi.org/10.1023/A:1006177414456

Weber, E., Engler, C., Gruetzner, R., Werner, S., & Marillonnet, S. (2011). A modular cloning system for standardized assembly of multigene constructs. *PLoS ONE*, *6*(2). https://doi.org/10.1371/journal.pone.0016765
